# Feasibility of bispectral index monitoring during pediatric critical care transport

**DOI:** 10.1111/pan.15000

**Published:** 2024-09-22

**Authors:** Adam Watson, Francesca Zanetti, Oliver Ross, John Pappachan, Michael J. Griksaitis

**Affiliations:** ^1^ Paediatric Intensive Care Unit University Hospital Southampton NHS FT Southampton UK; ^2^ Faculty of Medicine University of Southampton Southampton UK; ^3^ Department of Paediatric Anaesthesia University Hospital Southampton NHS FT Southampton UK

**Keywords:** audit, depth of anesthesia, critical care, PICU, NICU, equipment, anesthetic machines, equipment, monitors, non‐invasive, general anesthesia

To the Editors,

The majority of children undergoing pediatric critical care transport (PCCT) are sedated, intubated, and muscle relaxed, and therefore, there is a risk of accidental awareness,^1^ particular in adolescent children. Bispectal index (BIS) is a processed electroencephalography device which monitors depth of anesthesia. To our knowledge, no PCCT service in the world routinely uses or has investigated BIS monitoring during inter‐hospital pediatric critical care transport. Here, we describe the feasibility of BIS monitoring in our PCCT service.

Southampton Oxford Retrieval Team introduced BIS monitoring during PCCT on September 1, 2023, for sedated, intubated, and muscle relaxed children (term newborn to 16 years) being transported by road ambulance. Although BIS may be less meaningful in infants, we chose to include children of all ages as we aimed only to investigate device feasibility during PCCT. Our hospital routinely uses BIS during pediatric anesthesia and in intensive care, so as we sought only to extend our use of BIS to PCCT, this study met the criteria for service evaluation and the requirement for informed consent was waived. The PCCT team were blinded to BIS value and delivered usual care. BIS monitoring was commended upon arrival at referring hospitals and continued during road transport until arrival at the receiving PICU. BIS, signal quality index (SQI), and electromyography (EMG) data were classified as either “pre‐journey,” “traveling,” or “post‐journey.” The Kruskal–Wallis one‐way ANOVA with post hoc Dunn's testing was used to compare between these phases, and the Mann–Whitney *U*‐test to compare the 5 min before and after road travel started. We used SPSS v28 (IBM Corp., Armonk, NY) for our analysis, with a *p* value of <.05 taken as significant.

We obtained 1695 minutes of BIS, SQI, and EMG recordings from 27 children, with BIS monitoring unsuccessful in four further children (Table 1). The median age was 14 months (IQR 3–31), and median weight was 10.0 kg (IQR 5.3–13). Overall median BIS and SQI were 49 (IQR 42–61) and 94 (IQR 82–97) respectively, although BIS and SQI varied between phases of PCCT (*p* < .001). The median EMG was 30 (28–32), with no difference in EMG between phases of PCCT (*p* = .165). We compared between the 5 min before and after road travel started, with no difference in BIS (50 vs. 46, *p* = .358), SQI (91 vs. 92, *p* = .283), or EMG (29 vs. 29, *p* = .686).

**TABLE 1:** Bispectral index, signal quality index, and electromyography values during PCCT.

	Overall (*n* = 1695 min)	Pre‐journey (*n* = 308 min)	Traveling (*n* = 1218 min)	Post‐journey (*n* = 168 min)	*p* Value
BIS value					
Median (IQR)	49 (42–61)	50 (42–61)	47 (41–60)	60 (50–66)	<.001
Range	16–9	16–92	16–88	35–87	
SQI value					
Median (IQR)	94 (82–97)	89 (77–97)	94 (83–100)	94 (88–97)	<.001
Range	51–100	53–100	51–100	51–100	
EMG value					
Median (IQR)	30 (28–32)	30 (28–32)	30 (28–31)	29 (29–30)	.165
Range	18–50	19–42	18–50	22–50	

Abbreviations: BIS, bispectral index; EMG, electromyography; IQR, inter‐quartile range; SQI, signal quality index.

Our data suggest that in our PCCT service there is no significant effect of road transport on BIS, SQI, and EMG values, and therefore that these can be interpreted during PCCT as they would be for an equivalent hospital patient. However, this was a small single‐center study. Of importance, we do not believe that the ideal BIS range for children during PCCT is clear, and hypothesize that a median BIS of 49 could reflect a degree of oversedation. The use of density spectral array may help define this and may facilitate other benefits of BIS monitoring (e.g., seizure detection).^2^ Further work to investigate the utility and meaningfulness of BIS in a PCCT setting is needed, but our data suggest BIS is feasible during road transport.

## CONFLICT OF INTEREST STATEMENT

Medtronic Ltd. (Watford, UK) supplied us with two BIS monitors at no cost, but had no involvement with study delivery or manuscript preparation. We declare no other conflicts of interest.

## Data Availability

The data that support the findings of this study are available on request from the corresponding author. The data are not publicly available due to privacy or ethical restrictions.
